# Complement-Mediated Two-Step NETosis: Serum-Induced Complement Activation and Calcium Influx Generate NADPH Oxidase-Dependent NETs in Serum-Free Conditions

**DOI:** 10.3390/ijms25179625

**Published:** 2024-09-05

**Authors:** Maria Maqsood, Samuel Suntharalingham, Meraj Khan, Carolina G. Ortiz-Sandoval, Wouter J. C. Feitz, Nades Palaniyar, Christoph Licht

**Affiliations:** 1 Cell Biology, Research Institute, Peter Gilgan Centre for Research and Learning, The Hospital for Sick Children, Toronto, ON M5G 1X8, Canada; mmaqsood@mun.ca (M.M.); samuel.elijah.suntharalingham@gmail.com (S.S.); carolina.ortiz@sickkids.ca (C.G.O.-S.); wouter.feitz@radboudumc.nl (W.J.C.F.); 2 Translational Medicine, Research Institute, Peter Gilgan Centre for Research and Learning, The Hospital for Sick Children, Toronto, ON M5G 1X8, Canada; meraj.khan@sickkids.ca (M.K.); nades.palaniyar@sickkids.ca (N.P.); 3 Department of Pediatric Nephrology, Amalia Children’s Hospital, Radboudumc, 6525 GA Nijmegen, The Netherlands; 4 Department of Laboratory Medicine and Pathobiology, University of Toronto, Toronto, ON M5S 1A1, Canada; 5 Division of Nephrology, The Hospital for Sick Children, Toronto, ON M5G 1X8, Canada

**Keywords:** neutrophils, complement, NET formation, NETosis, citrullinated histone 3, P-selectin/CD11b

## Abstract

The complement system and neutrophils play crucial roles in innate immunity. Neutrophils release neutrophil extracellular traps (NETs), which are composed of decondensed DNA entangled with granular contents, as part of their innate immune function. Mechanisms governing complement-mediated NET formation remain unclear. In this study, we tested a two-step NETosis mechanism, as follows: classical complement-mediated neutrophil activation in serum and subsequent NET formation in serum-free conditions, using neutrophils from healthy donors, endothelial cells, and various assays (Fluo-4AM, DHR123, and SYTOX), along with flow cytometry and confocal microscopy. Our findings reveal that classical complement activation on neutrophils upregulated the membrane-anchored complement regulators CD46, CD55, and CD59. Additionally, complement activation increased CD11b on neutrophils, signifying activation and promoting their attachment to endothelial cells. Complement activation induced calcium influx and citrullination of histone 3 (CitH3) in neutrophils. However, CitH3 formation alone was insufficient for NET generation. Importantly, NET formation occurred only when neutrophils were in serum-free conditions. In such environments, neutrophils induced NADPH oxidase-dependent reactive oxygen species (ROS) production, leading to NET formation. Hence, we propose that complement-mediated NET formation involves a two-step process, as follows: complement deposition, neutrophil priming, calcium influx, CitH3 formation, and attachment to endothelial cells in serum. This is followed by NADPH-dependent ROS production and NET completion in serum-free conditions. Understanding this process may unveil treatment targets for pathologies involving complement activation and NET formation.

## 1. Introduction

Complement is a pattern-recognition system that can interconnect the adaptive immunity (e.g., antibodies) with the innate immune system (e.g., neutrophils, neutrophil extracellular traps, or NETs). The complement system can be activated through the following three distinct pathways: classical pathway (e.g., by recognizing multiple Fc regions of IgGs or IgMs immobilized on the targets), lectin pathway (e.g., by recognizing repeated terminal carbohydrate moieties present on microbial surfaces), and alternative pathway (constitutively active at a low rate on any surface). Regardless of the pathway of activation, all three pathways converge on the activation of C3 (e.g., opsonization), which can subsequently initiate the terminal pathway, leading to the formation of membrane attack complexes (MACs; C5b-9) to kill the microbial targets [1,2,3,4]. Our previous studies [5,6,7] and a few other studies [8,9] show that complement activation can deposit C3b and C5b-9 on neutrophil extracellular traps (NETs) or NET-bacteria complexes. Nevertheless, how complement activation on neutrophils regulate NET formation is not clearly understood.

Neutrophils are the major (60–70%) immune cells present in circulation. Upon activation, neutrophils undergo an increase in CD11b expression on their surfaces and shedding of CD62L (L-selectin). CD11b is an essential adhesion molecule that plays a crucial role in facilitating neutrophil–endothelial interactions and promoting neutrophil extravasation, allowing them to migrate from blood vessels to inflamed tissues [10,11,12]. Whether the complement activation on neutrophils can promote their interaction with endothelial cells in the context of NET formation is not well established.

Under specific stimulatory conditions, such as exposure to TNF-α, fMLP, PAF, or GM-CSF, neutrophils can undergo priming, which results in their activation and NETs release, composed of decondensed chromatin coated with cytotoxic granular proteins and enzymes, such as myeloperoxidase (MPO) [13,14,15]. Intracellular calcium and reactive oxygen species (ROS) play crucial roles in NET formation. ROS can originate from the following two primary sources in neutrophils: NADPH oxidase (NOX) or mitochondria [14,16,17]. Elevated intracellular calcium levels activate peptidylarginine deiminase 4 (PAD4), enabling the citrullination of histones to facilitate chromatin decondensation, independent of ROS generation [18]. These steps ultimately culminate in the decondensation of chromatin and NET formation. Suicidal NET formations (NETosis) are commonly classified into the following two categories: NOX-dependent and NOX-independent NETosis [14,18,19]. However, the role of intracellular calcium and type of ROS involved in complement-mediated NETosis are not clearly understood.

The presence of serum is known to inhibit ROS production by neutrophils and subsequent NET formation. Also, complement activation on host cells is regulated via soluble (e.g., Factor H [CFH]) and membrane-anchored (e.g., membrane cofactor protein [MCP/CD46], decay acceleration factor [DAF/CD55], complement receptor 1 [CR1/CD35], and protectin [CD59]) to efficiently target complement activation on foreign targets. However, clear evidence for the involvement of the classical pathway of complement in activating NETosis is limited. Here, we investigate the molecular and functional consequences of complement activation via the classical pathway on neutrophils and establish a two-step process of complement-mediated NET formation using well-defined experimental systems. The mechanistic understanding gleaned from this work could help to understand the stepwise complement-mediated NET formation events that can occur in the vasculature (serum) and in the extravascular tissue compartments (little or no serum). Hence, the procedures established and the key points uncovered in this study could help to conduct two-step experiments relevant to specific disease conditions involving complement system and NET formation.

## 2. Results

### 2.1. Classical Pathway of Complement Is Activated and Fixed on Anti-CD59 Antibody-Coated Neutrophils in Serum

**Figure 1:**
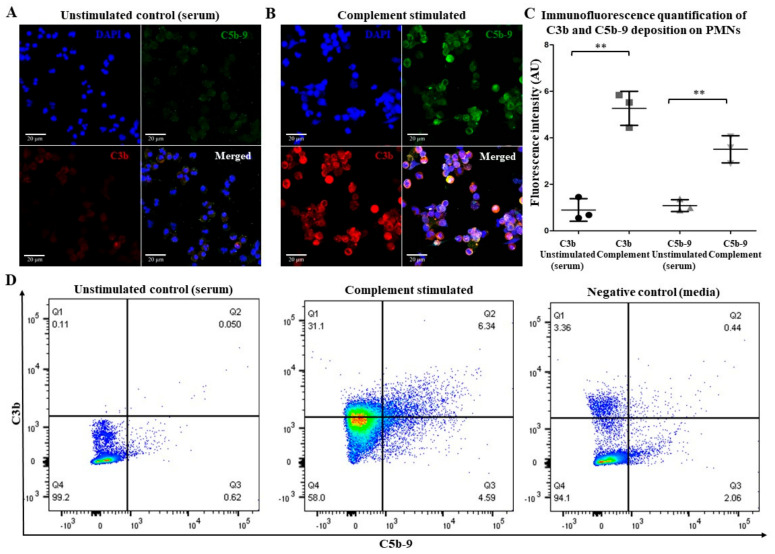
**Immunofluorescence microscopy and flow cytometry show that anti-CD59 IgG-mediated classical complement activation leads to C3b and C5b-9 depositions on neutrophils.** Neutrophils isolated from healthy donors were precoated with a monoclonal anti-CD59 antibodies, exposed to normal human serum, fixed and immunostained for C3b (red) and C5b-9 (green). (**A**) The confocal microscopy shows that C3b and C5b-9 were barely detectable in the control neutrophils. (**B**) The confocal microscopy shows strong staining for C3b and C5b-9 on antibody-coated neutrophils. (**C**) The quantifying fluorescence in the images shows that C3b and C5b-9 were significantly higher in the antibody-coated neutrophils than the control neutrophils. (**D**) The flow cytometry analyses also show that C3b and C5b-9 levels increased on complement-activated neutrophils compared to control neutrophils. Scale bar = 20 µm; 63× magnification. n = 3 biological replicates. ** *p* < 0.01, compared to their controls, based on the t-test. Data are presented as means ± SD.

To study complement activation on neutrophils, we used confocal immunostaining and flowcytometry assays. We incubated neutrophils with anti-CD59 and exposed them to normal human serum (NHS). This procedure is expected to recruit the classical pathway components and fix complement on the surface of neutrophils. We first immunostained these neutrophils with anti-C3b and examined them using immunoconfocal microscopy. The images showed the deposition of large amounts of C3b (red) on antibody-coated neutrophils but only marginally on control neutrophils (Figure 1A,B).

Quantifying the fluorescence intensity of C3b confirmed that significantly high amounts of complement was fixed by this procedure. Analyses of these neutrophils by flow cytometry confirmed the microscopy-based results. Hence, this experimental procedure effectively deposited complement on neutrophils. To further determine whether complement activation progresses and forms C5b-9 or MAC, we immunostained these cells with anti-C5b-9 antibodies. Both the image analyses and flow cytometry data show that the procedure led to the deposition of C3b and the formation of a significant amount of C5b-9 (green) on neutrophils (Figure 1A–D). Therefore, anti-CD59-mediated complement activation on neutrophils with NHS effectively activates and fixes complement on these cells, in serum.

### 2.2. Complement Activation Upregulates Complement Regulators on Neutrophils

**Figure 2:**
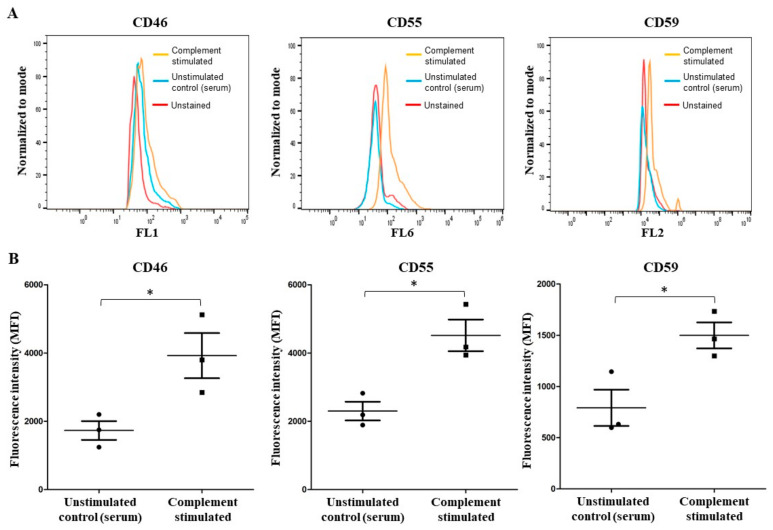
**Complement activation increases complement regulators on neutrophils.** Neutrophils were precoated with anti-CD59 monoclonal antibodies and then exposed to normal human serum (NHS) to activate the complement cascade (complement stimulated). The group not treated with anti-CD59 monoclonal antibodies served as the unstimulated control (serum). The cells were then fixed, immunostained, and analyzed by flow cytometry. The expression level of all three complement regulators (CD46, CD55, and CD59) was significantly increased on complement-activated neutrophils compared to the unstimulated controls. (**A**) Representative flow cytometry tracings of an experiment; (**B**) mean fluorescence intensity (MFI) for each marker from all experiments. n = 3 biological replicates. * *p* < 0.05, compared to their controls, based on the paired t-test. Data are presented as means ± SD.

The effects of complement activation on neutrophils on complement regulators are not clearly established. To determine the effect of complement activation on neutrophils, we immunostained control neutrophils and complement-activated neutrophils and examined them by flow cytometry. The analyses showed that complement activation increased the levels of membrane-anchored complement regulators, such as membrane cofactor protein (MCP/CD46), decay acceleration factor (DAF/CD55), and protectin (CD59), on complement-fixed neutrophils, compared to unstimulated controls (Figure 2). These results show that complement activation on neutrophils increases complement regulatory proteins on these neutrophils.

### 2.3. Complement Fixation Activates Neutrophils and Promotes Neutrophil–Endothelial Cell Interactions

**Figure 3:**
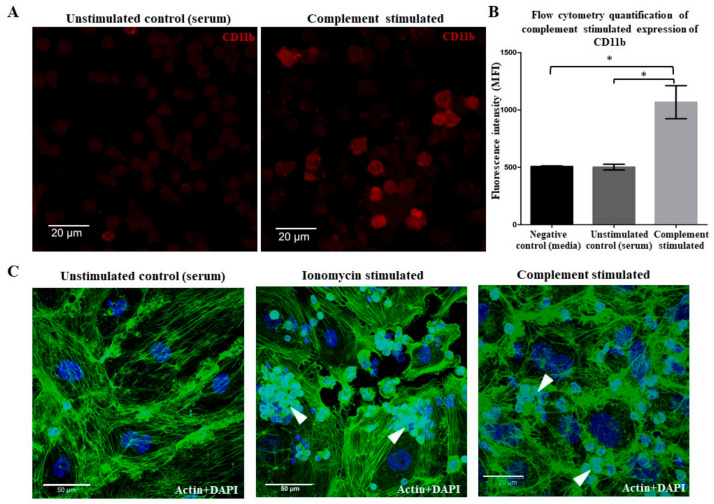
**Complement activation increases the activation marker CD11b on neutrophils.** Neutrophils were precoated with anti-CD59 monoclonal antibodies and then exposed to normal human serum (NHS) to activate the complement cascade (complement stimulated). The group not treated with anti-CD59 monoclonal antibodies served as the unstimulated control (serum). The cells were then fixed and immunostained for CD11b. (**A**) Immunofluorescence microscopy shows the increase in CD11b on complement-activated neutrophils compared to unstimulated controls, representative of the 3 experiments. Scale bar = 20 µm; 63× magnification. (**B**) Mean fluorescence intensity (MFI) of obtained from the flow cytometry experiments showing similar results. Complement-activated neutrophils increased the levels of CD11b, compared to unstimulated controls (serum or serum-free conditions). (**C**) The confocal microscopy shows that complement-activated or ionomycin-treated (positive control) neutrophils effectively inter-reacted with endothelial cells (incubated for 1 h and stained for DNA (DAPI, blue) and immunostained for actin (green)), compared to nonactivated control neutrophils. Arrowheads: neutrophils. Scale bar = 20 µm. Images were captured at 63× magnification. n = 3 biological replicates. * *p* < 0.05, compared to either control. Data are presented as means ± SD.

To determine whether complement fixation activates neutrophils, we measured CD11b surface levels using immunofluorescence microscopy and flow cytometry. We observed an increase in CD11b levels on the surface of complement-fixed neutrophils compared to the controls (Figure 3A,B), indicating the activation of neutrophils. Activated neutrophils are known to interact effectively with endothelial cells. Therefore, to determine whether neutrophils activated by complement activation interact with endothelial cells, we examined their interactions with BOECs. Confocal microscopy showed that complement-activated neutrophils (smaller cells, arrowheads) effectively adhered to the endothelial monolayer (larger cells, green actin fibers), compared to nonactivated neutrophils (Figure 3C). Together, these results show that complement activation on neutrophils leads to their effective interaction with endothelial cells. Furthermore, we performed a densitometry analysis of the immunostained images. Relative to the staining of the ionomycin set at 100%, the complement-stimulated samples showed a 69% fluorescence intensity, whereas unstimulated controls (i.e., serum) exhibited only 17%. This difference indicates an increased adherence of neutrophils under complement stimulation compared to serum controls. These quantitative data provide further support for our observations of cell–cell interactions in the experimental setup.

### 2.4. Complement Fixation Activates Certain Steps of NETosis in Neutrophils

**Figure 4:**
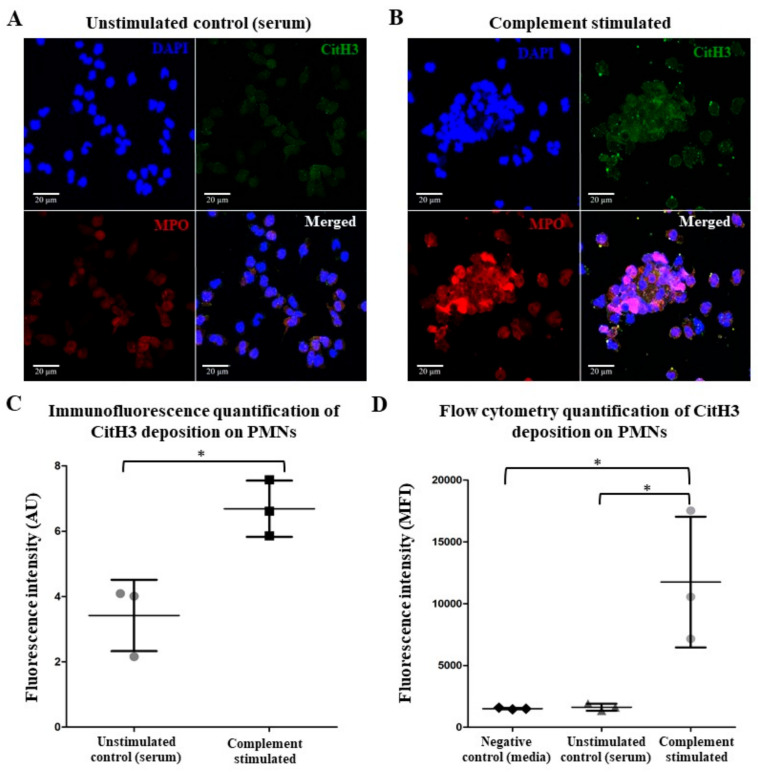
**Complement activation increases immunostaining for myeloperoxidase and citrullination of histones in neutrophils.** (**A**,**B**) Neutrophils without (**A**) or with (**B**) precoating of anti-CD59 antibodies were incubated with serum, fixed, and immunostained for myeloperoxidase (MPO) and citrullinated histone 3 (CitH3). The confocal microscopy shows that the MPO (red) and CitH3 (green) staining levels were higher in complement-activated neutrophils compared to unstimulated controls. (**C**) Quantified fluorescence of confirmed visual observations of CitH3. (**D**) The mean fluorescence intensity (MFI) of flow cytometry analyses also shows that CitH3 levels were higher on complement-activated cells compared to control neutrophils. Scale bar = 20 µm. Images were captured at 63× magnification. n = 3 biological replicates. * *p* < 0.05, compared to either control. Data are presented as means ± SD.

In the presence of serum, neutrophils often do not undergo NETosis [2,20]. However, whether complement activation on neutrophils in serum leads to NET formation is not well understood. To determine the effect of complement activation on the steps of NET formation, we examined the classical NETosis markers (e.g., MPO, citrullinated histone 3, or CitH3) on these neutrophils. Immunostaining showed that complement activation on neutrophils in the serum condition showed that these neutrophils remained intact but had increased CitH3 and MPO staining, often at the surface, compared to unstimulated controls (Figure 4A–C). Analyzing these neutrophils using flow cytometry also confirmed the microscopy data (Figure 4D). We previously published work on the immunostaining of NETs using CitH3 and MPO, with a well-established protocol featured in numerous studies incorporating various technical and biological controls [18,21]. Of note, we did not observe any precoating effect in the staining process. The decision to omit the “anti-CD59, no complement control” was made to concentrate our study specifically on the role of CD59 in complement activation.

**Figure 5:**
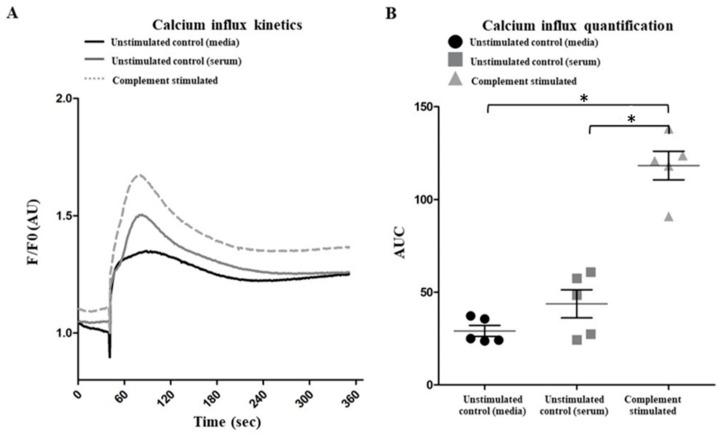
**Complement activation increases intracellular calcium concentration in neutrophils.** (**A**) Neutrophils were incubated with the Fluo-4 dye in the presence or absence of anti-CD59 antibodies, washed and placed in serum-free media (complement stimulated: neutrophils were precoated with anti-CD59 antibody and then exposed to NHS; unstimulated control (serum): not treated with anti-CD59 antibody; and unstimulated control (media): not treated, and no serum, only media added to the cells). Calcium levels in the cells were standardized to the initial reading (F/F0). Kinetic graphs indicate that complement activation increased intracellular calcium levels (representative of 5 experiments). (**B**) Area under the curve showing that the calcium influx into complement-activated cells is significantly higher than the control neutrophils placed in serum or serum-free media. n = 5 biological replicates. * *p* < 0.01, compared to controls. Data are presented as means ± SD.

Increased intracellular calcium concentration is a prerequisite for the citrullination of histones in neutrophils [21,22]. Therefore, we measured the intracellular calcium concentrations of these neutrophils using Fluo-4AM dye. Neutrophils subjected to complement activation had increased intracellular calcium, compared to nonactivated neutrophils (Figure 5). Therefore, complement activation increases intracellular calcium levels and promotes citrullination of histones in the neutrophils.

### 2.5. Complement-Fixed Neutrophils Undergo Full NETosis in Serum-Free Conditions

**Figure 6:**
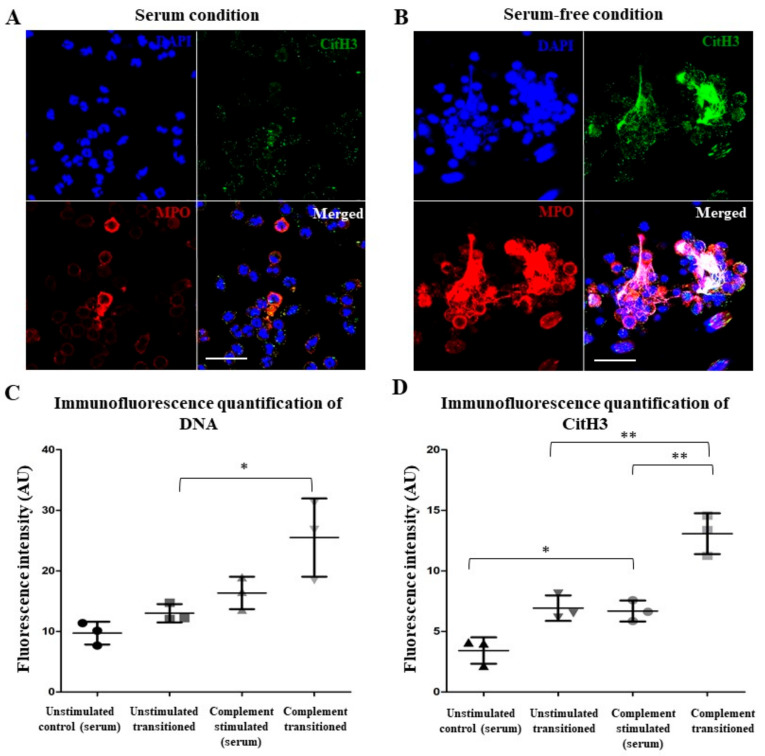
**Transitioning complement-activated neutrophils from serum to serum-free conditions induces NETosis.** (**A**,**B**) Fluorescence microscopy showing the effect of complement activation on NETosis during the transition from serum to serum-free conditions. Neutrophils were coated with anti-CD59 monoclonal IgG, incubated in serum, fixed, and immunostained for CitH3 and MPO, with or without transitioning to serum-free conditions. Higher degrees of CitH3 and MPO colocalization and extracellular traps were detected after the transition (**B**) than before the transition (**A**). Scale bar = 20 μm; 63× magnification. Representative of n > 3 (each replicate represents an independent experiment conducted using different healthy donors for neutrophil isolation). (**C**,**D**) Quantitative analyses of CitH3 and DNA images, confirming the observations shown in (**A**,**B**). One-way ANOVA with Dunnett’s post-test. * *p*-value < 0.05; ** *p*-value < 0.01. Data are presented as means ± SD.

**Figure 7:**
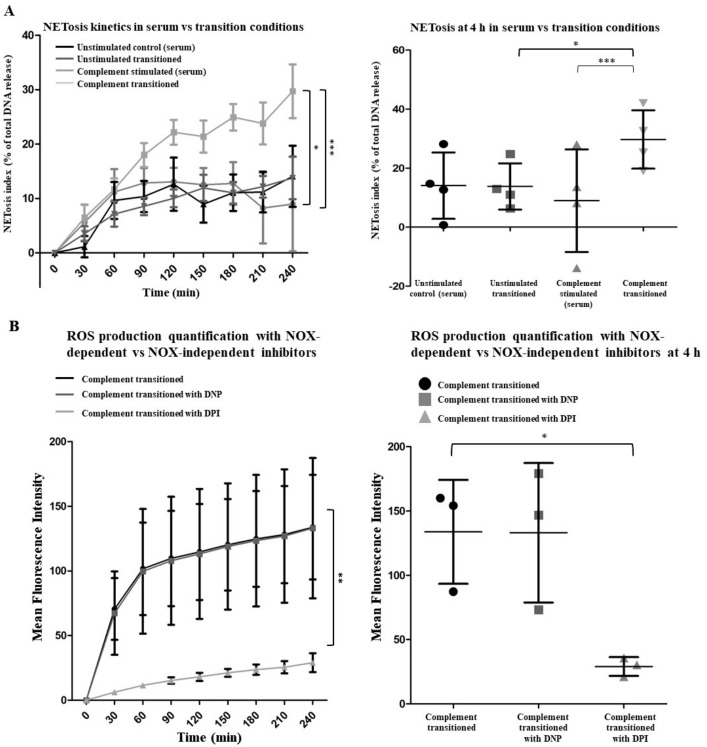
**Quantitative analyses show that the NOX-dependent pathway is involved in complement-mediated NET formation.** (**A**) The NET release kinetics were monitored by SYTOX kinetics assay. Complement-stimulated neutrophils release significantly higher amounts of NETs after transitioning from serum to serum-free conditions, compared to other conditions. n = 4 biological replicates; two-way ANOVA. *** *p*-value < 0.001; * *p*-value < 0.05. (**B**) Transferring the complement-activated neutrophils from serum to serum-free condition leads to an increase in ROS generated by NOX but not by mitochondria, as determined by the DHR123 assays. DPI (NOX inhibitor) but not DNP (mitochondrial uncoupler) significantly suppressed ROS generation, as determined by two-way ANOVA with repeated measure, overtime. Comparing the total ROS at a 4 h time point confirmed the differences observed in the kinetics data, as determined by one-way ANOVA with Dunnett’s post-test. * *p* < 0.05; ** *p* < 0.01. *n* = 3 biological replicates. Data are presented as means ± SD.

Neutrophils migrate to various tissues (e.g., glomeruli) that have little or no serum. However, whether complement-activated neutrophils undergo NETosis under serum-free conditions is not established. Hence, we transitioned the complement-activated neutrophils from the serum into serum-free media. Immunofluorescence microscopy images show that NET structures (DNA and DAPI blue) with MPO and CitH3 are readily visible after transitioning the complement-activated neutrophils into serum-free conditions (Figure 6A,B). Quantifying the fluorescence confirmed the qualitative image data (Figure 6C,D). To further understand the NETosis kinetics, we used a SYTOX assay. Neutrophils under all three control conditions (neutrophils in serum-free media, neutrophils in serum, nonactivated neutrophils transitioned from serum to serum-free conditions) had similar baselines. In contrast, transition of the complement-activated neutrophils from the serum into serum-free media allowed for NETosis to occur over time, compared to controls (Figure 7A).

ROS generation from NADPH oxidase (NOX) or mitochondria is a prerequisite for NETosis [14]. Hence, to determine the type of ROS responsible for complement-mediated NETosis, we used a NOX inhibitor diphenyleneiodonium chloride (DPI) or mitochondrial ROS inhibitor 2,4-dinitrophenol (DNP), and quantified the ROS produced by DHR123 assays. When the complement activated neutrophils were transitioned from the serum to serum-free conditions, these neutrophils generated ROS (Figure 7B). DPI, but not DNP, suppressed the complement-mediated ROS production. Therefore, complement-activated neutrophils generate ROS by NOX, indicating that complement-activated neutrophils undergo NOX-dependent NETosis in the serum-free condition.

## 3. Discussion

Understanding the roles of complement activation in neutrophil extracellular trap (NET) formation is important for elucidating the mechanisms of immune responses in various diseases. While it has been shown that various stimuli can induce NETosis in the absence of serum, the specific roles of complement activation in this process are not yet clearly defined. Previous research has suggested that complement can deposit on NETs and bacteria through the activation of the alternative pathway [7,8], but certain serum components, such as albumin [2,20,23] and complement factor H [24], can inhibit ROS production and potentially suppress NETosis. In this context, it is important to systematically establish the effect of classical complement activation on NETosis in the presence of serum. Our study provides insights into this process, showing that classical complement activation on antibody-coated neutrophils in the presence of serum leads to the deposition of C3b and eventual assembly of C5b-9 on neutrophils, as well as an increase in cell surface complement inhibitors and activation markers. These events, ultimately, prime neutrophils for NETosis and enhance their interactions with endothelial cells, and when primed neutrophils are transitioned to serum-free conditions, they generate ROS and undergo full NETosis. This two-step process provides unique mechanistic insights into complement-mediated NETosis and could have important implications for NET-related diseases (e.g., autoimmunity and complement-related damage to basement membranes in kidney).

The activation of complement on neutrophils can occur because of the anti-neutrophil antibodies present in the sera of certain auto-immune patients (e.g., ANCA vasculitis [8]. The classical pathway can also be activated on cells using purified antibodies and NHS [6,25,26,27,28]. Our data show that an antibody against neutrophil surfaces of CD59 and NHS can be used for activating the classical pathway of complement on neutrophils that leads to C3b deposition and subsequent C5b-9 complex formation via terminal pathway activation (Figure 1). Complement fixation occurs despite the increase in complement regulators (MCP/CD46, DAF/CD55, and protectin/CD55) on neutrophils (Figure 2). These results are consistent with previous studies showing the upregulation of DAF/CD55 in response to neutrophil activation [26]. Complement activation also increases CD11b (αM integrin chain) (Figure 3A,B) that complexes with CD18 (β2 integrin chain) to form the C3 complement receptor (CR3 or Mac-1) on neutrophils [29,30]. CD11b is a key molecule necessary for neutrophil interaction with endothelial cells and crawling. Such an increase could explain the increased interaction between complement-activated or ionomycin-treated neutrophils with endothelial cells (Figure 3C). This type of interaction could promote transmigration of these neutrophils to extravascular spaces.

Interestingly, complement activation on neutrophils present in the serum leads to the increased detection of MPO and CitH3 (Figure 4). Complement-activated neutrophils in the serum maintain their spherical morphology with no drastic changes to polymorphonuclear morphology. Previous studies have detected CitH3 and MPO on the NETosis-prone neutrophils, a phenomenon also described by Fine et al. in primed neutrophils from both humans and mice [2,11]. Citrullination of histones requires an increase in intracellular calcium concentrations allowing for the PAD4 enzyme to bind calcium, translocate to the nucleus, and deiminate arginine present on the histones to citrulline [18,31,32]. We also found that complement activation increases intracellular calcium (Figure 5). MAC formed during terminal pathway activation [27,33,34] and/or the engagement of C5a with C5a receptor could also promote calcium influx [35,36,37]. In one study, neutrophils stimulated with N-formylmethionine-leucyl-phenylalanine (fMLP) that were treated with SK&F 96,365 (an inhibitor of receptor-mediated calcium entry (RMCE)) decreased calcium influx by 50% but had little to no effect on internal calcium release [38]. Taken together, complement activation on neutrophils results in calcium influx and hypercitrullination like calcium-ionophore-mediated NETosis.

ROS are essential for NETosis [2,20,39]. Under the serum conditions, the ROS levels were suppressed in neutrophils. For example, albumin scavengers ROS and suppress NETosis [2,20], whereas during complement activation, FH binds to CD11b, degrades C3b, and suppresses ROS production and NETosis, although the mechanism is not fully understood [24]. However, complement-mediated NETosis was completed when these neutrophils were transferred from serum to serum-free media (Figure 6 and Figure 7A). This shift also changes the redox balance and removes the suppressive effect of serum components to allow the neutrophils to generate large amounts of ROS in these neutrophils (Figure 7B). ROS can be generated by NOX and mitochondria for NOX-dependent and NOX-independent forms of NET formation, respectively [21]. In the serum-free conditions, complement-activated neutrophils generated ROS via NOX and, hence, underwent a NOX-dependent NETosis (Figure 7B). Yousefi et al. showed that neutrophils primed with granulocyte/macrophage colony-stimulating factor (GM-CSF) and exposed to anaphylatoxin C5a generates NOX-dependent ROS production but release mitochondrial DNA within 15 min via a vital form of NETosis; both stimuli were needed for the ROS production and the vital NET formation [9]. We also showed that neutrophils stimulated with C5a alone neither generated ROS above baseline nor formed NETs [7]. Hence, the current study establishes a unique two-step complement activation on neutrophil (C3b and C5b-9 formation in the serum, calcium influx, and CitH3 formation) and subsequent NOX-mediated ROS production and NET formation.

Although ROS are essential for NET formation, its role in NET formation remains uncertain. Recent studies show that ROS-mediated oxidation of DNA bases and subsequent base excision repair steps leading to DNA nick formation facilitate the drastic chromatin decondensation necessary for NET formation [40]. DNA repair is often coupled to transcription. Khan and Palaniyar previously showed that a genome-wide transcription is needed for NETosis induced by NOX-dependent and NOX-independent NETosis [21]. We and others also showed that CitH3 formation is a calcium-dependent process, and partly contributes to chromatin decondensation and can occur independent of the type of NETosis [18]. Hence, complement-mediated NET formation also likely to follow intracellular mechanisms mediated by both CitH3 generated by the presence of high concentrations of intracellular calcium and ROS generated by the NOX.

This two-step NETosis could be relevant to many in vivo situations. For example, systemic complement activation in intravascular space could lead to neutrophil priming and allows for the adherence of these neutrophils to endothelial cells. Subsequently, these neutrophils can extravasate to extravascular spaces (e.g., sites of infection with chemokine production). Once extravasated, complement-activated neutrophils should undergo full NET formation in the serum-free extravascular space. Previous studies show that incubating activated neutrophils with serum or albumin decreases NETosis [2,20]. In addition to the direct cytotoxic effect, NET DNA present in the extracellular spaces can also act as a damage-associated molecular pattern (DAMP), leading to a proinflammatory response and organ failure [41].

The two-step complement-mediated NET formation described in this study is likely to be relevant to clinically relevant disease conditions. NETs are being identified in many complement-mediated kidney diseases [12,42,43,44,45,46,47,48]. We recently demonstrated that complement activation on endothelial cells leads to neutrophil–platelet aggregate formation on endothelial cells [6]. Others show that suppression of terminal pathway by C5aR antagonism is beneficial both in vivo and in patients with ANCA-vasculitis [8]. Taken together, the current study suggests that the two-step NET formation is a functional consequence of complement activation on neutrophils and calcium influx in the serum condition, and NOX-dependent ROS production and NETosis completion in serum-free conditions.

## 4. Materials and Methods

### 4.1. Chemicals and Reagent

Unless indicated differently, all reagents were obtained from Sigma Aldrich (St. Louis, MO, USA).

### 4.2. Neutrophil Isolation

Whole blood was drawn from healthy male donors in EDTA (ethylenediaminetetraacetic acid) vacutainer tubes (Becton, Dickinson and Co, Franklin Lakes, NJ, USA; BD 367,856) to prevent clotting. Neutrophils were isolated using differential centrifugation by layering whole blood onto PolymorphPrep solution (Alere Technologies AS, Kjelsåsveien 161, 0884 Oslo, Norway; Cat # XS-1114683) at a 1:1 volume ratio (equal parts blood equal parts PolymorphPrepTM) (600 rcf for 35 min; 0 accel/deaccel). The layer of neutrophils was washed with a 0.4% NaCl + 10 mM HEPES (4-(2-hydroxyethyl)-1-piperazineethanesulfonic acid (Wisent, QC, Canada; 330-050-EL) solution. To lyse any residual red blood cells (RBCs) the PMN layer was washed with a hypotonic solution of 0.2% NaCl for 30 s and then neutralized using a 1.6% NaCl + 20 mM HEPES solution. The remaining cellular debris was washed using 0.85% NaCl and the cells were resuspended in Roswell Park Memorial Institute (RPMI) 1640 medium (Wisent, QC, Canada; 350-046-CL) supplemented with 10 mM HEPES buffer. The concentration and quality of neutrophils was determined trypan blue method using a hemocytometer (Wisent, QC, Canada; Cat# 609-130-EL). The purity of the neutrophils was determined by Cytospin preparations. Neutrophil preparations with 95–98% live and pure were used in all the experiments, reported in this study.

### 4.3. PMN Activator/Inducer Preparation

#### 4.3.1. Known PMN Activators/Inducers

All chemical and nonchemical inducers were bought from Sigma Aldrich (St. Louis, MO, USA) and prepared according to the manufacturer’s instructions. Inducers stock solutions were prepared in 1% DMSO and individual stock stored in −20 °C. The stock solutions were diluted in PBS to a final working concentration of 5 µM for Ionomycin (cat# I9657), 5 µg/mL for LPS (Lypopolysaccharide *E. coli* O128:B12; Cat# L2887), and 25 nM for phorbol 12-myristate 13-acetate (PMA) (cat# P1585) to stimulate neutrophils. The same concentration of DMSO has been used in media control to nullify the effect of DMSO in inducers conditions.

#### 4.3.2. Complement Sensitization Protocol

Neutrophils stimulated with complement were incubated with a monoclonal antibody against complement regulator, CD59 (goat anti-CD59, R&D Systems, Minneapolis, MN, USA, Cat# AF1987) reconstituted in PBS, at a concentration of 5 µg/mL for 30 min in serum-free RPMI media at 37 °C and 5% CO_2_. The cells were then exposed to 50% normal human serum (NHS) in serum-free media (SFM) to expose them to complement proteins to complete the cascade. In the transfer conditions, the neutrophils were then washed from this serum-containing media and replaced with SFM after an hour incubation.

### 4.4. Collection of Human Serum

Whole blood (30–60 mL) was collected from adult healthy volunteers in red cap vacutainer tubes with clot activator (silica particles) (Becton Dickinson, Franklin Lakes, NJ, USA; Cat# BD367812). The collected blood allowed to sit/clot for 30 min and was then centrifuged at 1500 rpm for 10 min to separate the serum. Sera of n = 3–5 healthy donors were pooled and stored at −20 °C until required for experimentation.

### 4.5. Neutrophil Immunofluorescence via Confocal Laser Microscopy

A solution of 2 × 10^6^ cells/mL (100 µL) neutrophils were seeded in a 12-well chamber slide. According to the environmental condition, either 100 µL of pooled 50% (*v*/*v*) NHS or SFM was added to each well along with the corresponding agonist to stimulate the neutrophils. The cells were incubated for either 4, 8, or 12 h (specific time points per experiment mentioned in corresponding figure caption) at 37 °C and 5% (*v*/*v*) CO_2_ to test for their activation. For the transition condition, stimulated cells were incubated in 50% (*v*/*v*) NHS plus 50% serum-free RPMI media containing 10 mM HEPES for 1 h at 37 °C and 5% (*v*/*v*) CO_2_ and then washed into 100% serum-free RPMI media with 10 mM HEPES for the rest of the incubation period. Complement stimulated neutrophils showed signs of priming and NET formation as early as 8 h and continued to show activation, to a higher extent, in the 12 h time point. Neutrophils were fixed using 16% paraformaldehyde (working concentration of 4%, *w*/*v*) in the wells, overnight. After incubation, the cells were washed with PBS and permeabilized with a 0.01% (*v*/*v*) Triton X-100 for 10 min at room temperature. Blocking from nonspecific antigen binding was done using Image-iT TM FX Signal Enhancer (ThermoFisher Scientific, Waltham, MA, USA; I36933) for 1 h at room temperature. In conditions examining PMN activation, neutrophils were washed and incubated with primary antibody against myeloperoxidase (MPO; Cat# ab25989, Abcam, Cambridge, UK; 1:250 dilution) overnight and a secondary antibody conjugated with Alexa Fluor 555 dye (ThermoFisher Scientific, Waltham, MA, USA; 1:500 dilution) for 45 min; and citrullinated histone 3 (CitH3; Cat# ab5103, Abcam, Cambridge, UK; 1:500 dilution) and a secondary antibody conjugated with Alexa Fluor 488 dye (ThermoFisher Scientific, Waltham, MA, USA; 1:500 dilution). In conditions examining complement deposition, neutrophils were washed and incubated with primary antibody against C3b (ThermoFisher Invitrogen, MA1-70054; 1:500 dilution) overnight and a secondary antibody conjugated with Alexa Fluor 555 dye (Thermo Fisher Scientific; 1:500 dilution) for 45 min; and C5b-9 (Cat# Abcam Ab55811; 1:250 dilution) and a secondary antibody conjugated with Alexa Fluor 488 dye (Thermo Fisher Scientific; 1:500 dilution). DNA was stained using 0.12 µg/mL Hoechst 33,342 stain (Cat# H3570, Invitrogen, Life Technologies, Carlsbad, CA, USA) for 1 h. After washing, the remaining media was removed, and the slides were mounted with a glass cover slip using a fluorescence mounting medium DAKO (Agilent Technologies, Santa Clara, CA, USA; Cat# S302380-2) at 1:1000 dilution. All images were captured using a Zeiss LSM880 AiryScan confocal laser scanning microscope. The images were taken at 63 X magnification and processed by the AiryScan processing software (ZEN v 3.8). Captured images were used to quantify the fluorescence intensity of the stained target by using ImageJ software (ImageJ 1.53t). Fluorescence intensities of individual targets were estimated by scanning more than 5 images retrieved from different experimental repeats under the consistent settings of the RGB and exposure.

### 4.6. Flow Cytometry

Neutrophils at a concentration of 1 × 10^6^ cells/tube were stimulated with our established complement sensitization protocol and incubated at 37 °C and 5% (*v*/*v*) CO_2_ for 1, 2, 3 or 4 h. The cells were then fixed in paraformaldehyde (4%, *w*/*v*) for 30 min and washed. Depending on the experiments performed, cells were either permeabilized with 0.01% (*v*/*v*) Triton-X 100 for 20 min at room temperature or remained nonpermeabilized to keep measurements to the surface of the cell. We used Heat Inactivated Serum (HIS) as one of our negative controls in the flow cytometry experiment, in the same way as stated in other figures. To specifically deactivate the complement activity of serum, while leaving other serum proteins functional, serum was heated for 56 degrees Celsius for 30 min. Primary antibodies C3b (1:500; Mouse, ThermoFisher Invitrogen, Carlsbad, CA, USA; Cat# MA1-70054), C5b-9 (1:250, Rabbit, Abcam, Toronto, ON, Canada; Cat# Ab55811), CD46 (1:200, Rabbit, Santa Cruz Biotech, Dallas, TX, USA; Cat# SC 9098), CD55 (1:250, Goat, R&D Systems, 614 McKinley Place NE, Minneapolis, MN, USA; Cat# AF2009), CD59 (1:200, Rabbit ThermoFisher Invitrogen, Carlsbad, CA, USA; Cat# PA5-34513), and PE CD11b (BioLegend, Markham, ON, Canada; Cat# 301306) and were incubated for 30 min on dark ice and then rinsed twice with spins at 400 g for 10 min. Secondary antibodies, Alexa Fluor 555 dye (ThermoFisher Scientific, Waltham, MA 02451, USA; 1:500 dilution), Alexa Fluor 488 dye (ThermoFisher Scientific, Waltham, MA 02451, USA; 1:500 dilution), and Alexa Fluor 647 (ThermoFisher, Waltham, MA 02451, USA, Cat# A21447) for 45 min were incubated for 45 min in the dark on ice, followed by two rinses for 10 min each.

A minimum of 100,000 live cells were collected using a Beckman Coulter Gallios flow cytometer (Beckman Coulter, Brea, CA, USA), equipped with 4 excitation lasers (405 nm, 488 nm, 561 nm, and 633 nm). Data were analyzed with Kaluza software version 2.2 (Beckman Coulter). Fluorescence minus one (FMO) control were used in panels involving more than 2 concurrent stains in each panel to ascertain that gates for positive and negative populations were reliably put in place. Otherwise, single-colored controls (containing only one stain) along with an unstained control (containing no stains, accounting for autofluorescence) were used for setting such gates.

Single-color flow cytometry gating is a basic approach to analyze and quantify a specific cell population based on a single fluorescent parameter. Here, in these experiments, we used the single-colored controls (containing only one stain) along with an unstained control (containing no stains, accounting for autofluorescence) and calibrated the instrument by choosing scatter parameters, such as forward scatter (FSC) or side scatter (SSC), and gated the on the cell population of interest. This gate helped exclude debris and noncellular events. Furthermore, create a density plot of the fluorescence intensity of the single-color parameter. Visualized the distribution of events and identified the positive and negative populations. After gating and instrument calibration, events were captured for each sample prepared on the same batches of experiments. All events were exported and analyzed in FlowJoTM 7.0 for Windows (Ashland, OR, USA: Becton, Dickinson and Company; 2019) in which gates were reapplied identically to all analyzed samples per given experimental acquisition on flow cytometry.

All events ungated were exported and analyzed in FlowJoTM 7.0 for Windows (Ashland, OR, USA: Becton, Dickinson and Company; 2019) in which gates were reapplied identically to all analyzed samples per given experimental acquisition on flow cytometry.

### 4.7. Endothelial Cell Culture

Blood outgrowth endothelial cells (BOECs) were isolated from peripheral blood of 2 healthy adult volunteers, as previously described [5,49], and cultured in endothelial cell growth medium (Endothelial Cell Growth Medium 2 Kit (PromoCell, Heidelberg, Germany), 10% (*v*/*v*) fetal bovine serum (FBS; Wisent, QC, Canada; Cat# 080-150), and 1% (*w*/*v*) Antibiotic–Antimycotic (Wisent, QC, Canada; Cat# 450-115-EL). Cells were maintained at 37 °C in an environment with 5% CO_2_. Passages 3–12 were used. BOECs were allowed to grow until 80% confluency, and we then seeded on 6-well plates overnight. The following day, the neutrophils were isolated and activated using the abovementioned sensitization protocol by first incubating them with anti-CD59 antibody for 30 min and then exposing them to 50% NHS in SFM. Once the NHS was added, the stimulated neutrophils were added to the confluent layer of BOECs and allowed to incubate at 37 °C with 5% (*v*/*v*) CO_2_ for 1 h. The cells were then washed three times using PBS and fixed with paraformaldehyde (working concentration of 4%, *w*/*v*) at room temperature for 20 min. After fixing, the cells were washed three times with PBS and incubated with actin (Actin Green, Fisher; Waltham, MA 02451, USA, Cat# R37110) for 30 min. The cells were again washed, and DNA was stained using Hoechst 33,342 (0.12 µg/mL) for 10 min. After washing, the remaining media were removed, and the slides were mounted with a glass cover slip using a fluorescence mounting medium DAKO (1:1000 dilution). All images were captured using the Zeiss LSM880 Airyscan confocal laser scanning microscope (Zeiss, Oberkochen, Germany). The images were taken at 63× magnification and processed by the AiryScan software (ZEN 3.8.).

### 4.8. Calcium Influx Assay

A solution of 1 × 10^6^ cells/mL (50 µL) neutrophils was loaded with Fluo-4AM dye (FisherScientific, Richmond, ON, Canada: Cat# F14201) at a final working concentration of 2 µM for 30 min in medium with or without anti-CD59 antibodies. The cells were then washed with RPMI 1640 medium (Invitrogen, Carlsbad, CA, USA), supplemented with 10 mM HEPES buffer. The cells were seeded onto a 96-well plate and read using the automated plate imager Cytation 1 (BioTek, Winooski, VT, USA). The device Cytation 1 was coupled to an automatic dispenser that injected a solution of SFM with no agonist (negative control) or SFM with ionomycin (positive control) to ensure there was no background activation. Under the complement conditions, stimulated neutrophils were automatically exposed to 50% (*v*/*v*) NHS to provide active complement proteins. Images were taken every second for six minutes and analyzed by calculating the average fluorescence per frame per time point in each well. The data were normalized to the baseline fluorescence intensity (F0) and reported as means.

### 4.9. DHR Assay

To detect cytosolic ROS production, a kinetics experiment was conducted using a fluorescent indicator of cytosolic ROS, called dihydrorhodamine (DHR) 123 (Life Technologies, Carlsbad, CA, USA). A solution of DHR (10 µM) was prepared in DMSO. A solution of neutrophils, prepared at a concentration of 2 × 10^6^ cells per ml, was exposed to our complement activation protocol by first sensitizing with an anti-CD59 antibody for 30 min and then exposing the cells to 50% (*v*/*v*) NHS for an hour. The cells were then transitioned into serum-free media preincubated with DHR dye either in the presence or absence of a NOX-independent inhibitor, 2,4-dinitrophenol (DNP: mitochondrial ATP uncoupler inhibiting the mitochondrial ROS) (at final concentration of 750 µM), or a NOX-dependent inhibitor, diphenyleneiodonium chloride (DPI) (at a final concentration of 1 µM), purchased from Sigma Aldrich (St. Louis, MO, USA). Fluorescence measurements were taken every 30 min to monitor ROS production (excitation/emission: 504/523 Thermo Scientific Varioskan LUX Multimode Microplate Reader: Waltham, MA 02451, USA). A fluorescent measurement was taken once, 30 min before the addition of any agonist or treatment to establish a background baseline, and then every 30 min for 4 h. To calculate the ROS production, the baseline fluorescence measurement was subtracted from each time point.

### 4.10. NET Formation Kinetics Assay

To quantify the release of extracellular DNA, a real-time kinetics experiment was performed using a cell impermeable DNA-binding dye, called SYTOX Green (Life Technologies, Carlsbad, CA, USA; S7020). A working concentration of 5 µM Sytox Green was added in media (RPMI 1640 medium supplemented with 10 mM HEPES) containing unstimulated neutrophils prepared at a concertation of 1 × 10^6^ cells per ml and seeded into a 96-well plate with a total volume of 100 µL in each well. Fluorescence measurements were taken every 30 min to monitor PMN activation via the release of extracellular DNA (excitation/emission: 504/523; Thermo Scientific Varioskan LUX Multimode Microplate Reader). A measurement was taken once 30 min before the addition of any agonist or treatment to establish a background baseline. To calculate the % NET formation, the baseline fluorescence measurement was subtracted at each time point and divided by the total fluorescence considered as 100% (by the condition in which cells were stimulated with 1% [*v*/*v*] Triton X-100).

### 4.11. Statistics and Graphics

Data are presented as means ± SD. For kinetics experiments, statistical analyses were accomplished using two-way analysis of variance (ANOVA) with Bonferroni post-tests, unless otherwise stated. The unpaired Student’s *t*-test was used for two-group analyses, comparing the means of two independent groups. A *p*-value < 0.05 was set for statistical significance. All statistical analyses were performed using GraphPad Prism statistical analysis software (GraphPad Software Version 6.0, La Jolla, CA, USA). Adobe illustrator was also used for generating figures and diagrams.

## 5. Conclusions and Contribution to the Field

Our results point toward the functional interaction of two central components of innate immunity, neutrophils, and the complement system. This interaction might contribute to the pathology of several diseases lacking specific treatments and being associated with poor outcomes including systemic lupus erythematosus (SLE) and rheumatoid arthritis (RA). Our research advances the understanding of the interaction between these two innate immune responders and how their interaction might contribute to disease pathogenesis. Specifically, our results suggest that the complement mediated neutrophil activation lead the formation of NOX-dependent NETosis. In addition, we identify a stepwise process of complement induced NET formation depending on the presence of serum. Delineating the mechanism of complement induced NET formation in more detail and associating it to specific diseases will allow for the application of specific treatments with the potential of fundamentally improving patient outcomes.

## Data Availability

The original contributions presented in the study are included in the article, further inquiries can be directed to the corresponding author.
